# Multi-Target Recognition of Bananas and Automatic Positioning for the Inflorescence Axis Cutting Point

**DOI:** 10.3389/fpls.2021.705021

**Published:** 2021-11-02

**Authors:** Fengyun Wu, Jieli Duan, Siyu Chen, Yaxin Ye, Puye Ai, Zhou Yang

**Affiliations:** ^1^College of Engineering, South China Agricultural University, Guangzhou, China; ^2^Guangdong Provincial Key Laboratory of Conservation and Precision Utilization of Characteristic Agricultural Resources in Mountainous Areas, Jiaying University, Meizhou, China

**Keywords:** fruit detection, computer vision, recognition and localization, multi-feature classification, edge detection, vision sensing

## Abstract

Multi-target recognition and positioning using robots in orchards is a challenging task in modern precision agriculture owing to the presence of complex noise disturbance, including wind disturbance, changing illumination, and branch and leaf shading. To obtain the target information for a bud-cutting robotic operation, we employed a modified deep learning algorithm for the fast and precise recognition of banana fruits, inflorescence axes, and flower buds. Thus, the cutting point on the inflorescence axis was identified using an edge detection algorithm and geometric calculation. We proposed a modified YOLOv3 model based on clustering optimization and clarified the influence of front-lighting and backlighting on the model. Image segmentation and denoising were performed to obtain the edge images of the flower buds and inflorescence axes. The spatial geometry model was constructed on this basis. The center of symmetry and centroid were calculated for the edges of the flower buds. The equation for the position of the inflorescence axis was established, and the cutting point was determined. Experimental results showed that the modified YOLOv3 model based on clustering optimization showed excellent performance with good balance between speed and precision both under front-lighting and backlighting conditions. The total pixel positioning error between the calculated and manually determined optimal cutting point in the flower bud was 4 and 5 pixels under the front-lighting and backlighting conditions, respectively. The percentage of images that met the positioning requirements was 93 and 90%, respectively. The results indicate that the new method can satisfy the real-time operating requirements for the banana bud-cutting robot.

## Introduction

Recent years have seen an unprecedented rise in the cost of human labor, with the increase reaching up to 12–15% in 2019 (Fu et al., 2020). At present, banana buds are generally cut and picked manually, and the labor cost accounts for approximately 34–40% of the total cost of banana production. Moreover, labor shortage and an aging labor pool pose barriers to the development of the banana industry. Considering the above, mechanization and intelligentization of banana bud cutting and picking represent an inevitable development trend for the banana industry. In a banana plant, the buds are connected to the fruits via the inflorescence axes. At the intermediate middle stage of development, the buds need to be cut off manually to ensure the quality of the fruit. Developing vision-based bud-cutting robots capable of automatic perception and intelligent decision-making is important for reducing labor costs and building intelligent banana orchards.

In the fruit industry, visual inspection and image processing for the recognition and positioning of fruits and flowers are among the most intensively studied topics (Gongal et al., 2015; Stein et al., 2016; Tang et al., 2020). Visual features are used to differentiate between the targets and other objects (Saedi and Khosravi, 2020). Classical image processing algorithms include those based on color, threshold segmentation, and edge detection. These algorithms are generally used for determining fruit type and yield, positioning, and harvesting (Patricio and Rieder, 2018). Wu et al. (2019) developed an automatic tomato harvesting method that integrated multi-feature fusion and double-level classification. Oppenheim et al. (2017) detected and counted yellow tomato flowers using an unmanned aerial vehicle (UAV). The tomato flowers were detected and segmented using self-adaptive global thresholding, HSV color space segmentation, and the morphological method (Oppenheim et al., 2017). Wang et al. (2018) employed the color thresholding technique for image processing. They used the conventional pixel segmentation method to separate mango spike pixels from the crown (Wang et al., 2018).

Target fruit recognition and positioning in a field is quite challenging (Wang et al., 2017; Chen et al., 2021). The environment is complex, with constantly varying light conditions; the fruits, leaves, stems, or other targets may be shaded (Feng et al., 2019). For this reason, simple color-based or thresholding methods may not be suitable for target recognition in a field (Li et al., 2020). Machine learning (ML) emerged along with big data technology and high-performance computing, and is defined as a scientific field that allows machines to learn without rigorous programming. Traditional ML algorithms include decision trees, clustering, Bayesian classification, SVM, Adaboost, and so on. In recent decades, ML has been widely used in various fields of agriculture (Tsaftaris et al., 2016; Maione and Barbosa, 2019; Chopra et al., 2021; Wan Nurazwin Syazwani et al., 2021; Yoosefzadeh-Najafabadi et al., 2021). “Deep learning” is strongly related to the “neural network” in machine learning. Since detection algorithms based on ML require proper feature vector design for classification, and then use the feature vectors to extract pixel or super pixel features for classification to achieve detection, which strongly relies on personal prior knowledge and is rather difficult. Therefore, the use of deep convolutional neural networks for fruit and vegetable detection has received much attention in recent years. Compared with conventional color- and threshold-based models and manual feature extraction, neural networks have been proven successful in target fruit detection and positioning (Gongal et al., 2015). For deep learning, the target detection model can be repeatedly trained using different convolutions to mine deep-level features. Thus, the model can more properly detect the target fruits and vegetables in an uncontrollable field environment regardless of the lighting conditions.

The object detector used for deep learning algorithms is mainly divided into two types: one is a two-stage detector based on candidate regions, and the other is a single-stage detector based on the regression method. The first type of two-stage detector divides object detection into stages, as in the case of the R-CNN series. This type of neural network has been widely applied to deep learning for fruit and flower detection in orchards. Jia et al. (2020) proposed a modified Mask R-CNN architecture to detect apples. The accuracy of feature extraction obtained by combining the ResNet and DenseNet architectures was 97.31% (Jia et al., 2020). Sa et al. (2016) detected sweet peppers using the modified faster R-CNN algorithm, and the F1-score was 0.83 (Sa et al., 2016). Dias et al. (2018a) applied the CNN architecture to extract features from the candidate flower regions separated by superpixel segmentation. Subsequently, a support vector machine (SVM) was employed to detect apple flowers. Both the recall and the accuracy exceeded 90% (Dias et al., 2018a,b). They also used the fully convolutional network (FCN) to detect flowers from the images of apples, peaches, and pears (Dias et al., 2018b). Lin and Chen (2018) described a strawberry flower detector. The results showed that the deep-level faster R-CNN could effectively monitor strawberry flowers under different camera views, flower distances, overlaps, complex background lighting, and blurred lighting (Lin and Chen, 2018). Tian et al. (2020) proposed a modified Mask-R-CNN for a real case of apple flower segmentation, with the accuracy reaching 96.43%. Complex noises in field environments have considerable bearing on the precision of detection.

To rapidly classify and recognize the objects, researchers have put forward the second type of one-stage object detection algorithm. You only look once (YOLO) and single-shot multibox detector (SSD) series belong to this type (Redmon and Farhadi, 2017; Yin et al., 2020). Compared to two-stage object detectors, single-stage object detectors are faster. Koirala et al. (2019) compared the performance of six deep learning frameworks in mango detection, and the MangoYOLO architecture was constructed. The F1 score was 0.97, and the mean accuracy was 0.98. The elapsed time for detecting each image was 70 ms (Koirala et al., 2019). Santos et al. (2020) detected grapes using three networks, namely, Mask R-CNN, YOLOv2, and YOLOv3, with the F1-score being 0.91. Zhang et al. (2021) described a modified SSD detector based on fruit color and morphological features. The frame rate of the stereo depth camera for detecting palm fruits, durian fruits, and pineapples reached 16.71 frames per second (Zhang et al., 2021). Wang et al. (2021) described a modified YOLOv3-Litchi model for detecting densely distributed lychee fruits in a large visual scene, where the mean precision was 87.43%. Wu et al. (2020) reported a real-time apple flower detector method using the channel-pruned YOLOv4 deep learning algorithm, which had an mAP of 97.31%. The detection speed was 72.33 f/s (Wu et al., 2020).

For edge detection and cutting point determination for targets in orchards, Luo et al. (2018) recommended using visual positioning to determine the picking point (for cutting off the fruit axis) on the fruit axis of grapes. Zou et al. (2012) and Xiong et al. (2018) studied the positioning and error analysis of the picking point in the pedicel of litchi. Many methods for picking point positioning have been proposed for different fruit-picking robots. However, picking point positioning may be influenced by some factors. Given the variability of fruit type, size, shape, and color, it is almost impossible to design a universal image segmentation algorithm, which otherwise affects the determination of the image centroid of fruits.

In banana orchards, machine vision and deep learning algorithms have been successfully applied to the classification and detection of a single target, such as fruits or stems. Neupane et al. (2019) detected and counted banana plants from RGB aviation images collected by UAV using fast-RCNN. Banana fruits and flower buds are objects of multi-target detection in banana orchards. However, the flower buds and inflorescence axes do not always point vertically downwards. This image feature adds to the complexity and computational difficulty. The purpose of target classification and recognition from images is to realize behavioral control of the visual robot operation. One of the important considerations is balancing the accuracy and speed of multi-target detection (Soviany and Ionescu, 2019). Our research team detected the fruit axes of bananas using the Deeplab v3+semantic segmentation network. A multi-view 3D perception of the center of the fruit axis of bananas in a complex orchard environment was conducted (Chen et al., 2020). A YOLOv4 neural network was then used to extract deep-level features from the banana fruits, thus realizing the accurate detection of bananas of varying sizes (Fu et al., 2020). Determining the cutting points in the banana flower buds and inflorescence axes is a necessary prerequisite for decision-making in bud-cutting robots. Acquiring visual information regarding the cutting point is a technical difficulty due to wind disturbance, changing lighting conditions, and branch and leaf shading.

Addressing complex environmental noises, we modified the YOLOv3 algorithm for accurate and rapid multi-target classification and recognition, Some parameters in YOLOv3 are optimized, and on the basis of clustering algorithm, the cross entropy loss function is introduced into the confidence and classification error model. An image segmentation and denoising algorithm was used to obtain the images of banana buds and inflorescence axes. A spatial geometry model was thus established. The function for positioning the inflorescence axis was based on finding the centroid of the banana bud. The coordinate information of the cutting point was obtained.

The highlights of the present study mainly include the following:

(1) Images of banana buds and inflorescence axes were obtained by image segmentation and denoising. The center of symmetry and the centroid were calculated for the edges of the flower buds.
(2) The cutting point was positioned using an edge image processing algorithm and the geometric method. Thus, the function for solving the inflorescence axis was established.

## Materials and Data Collection

### Testing Equipment and Software

**FIGURE 1:**
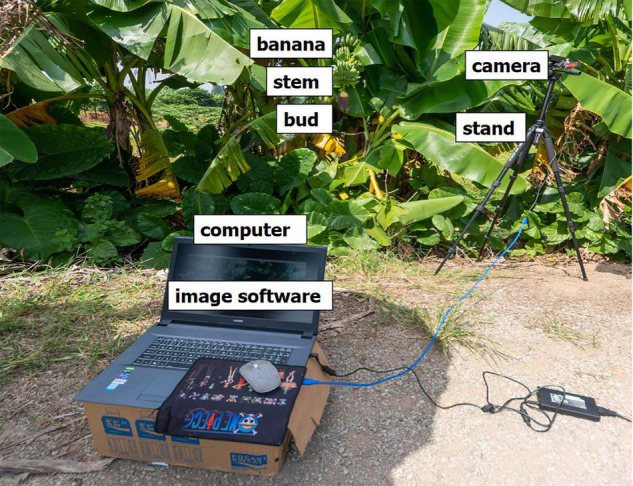
Computer, software, and camera.

The testing equipment had both hardware and software components. The hardware part of the testing equipment (Figure 1) included a computer for image processing, with the following configuration: i7-7700K processor, memory 16G, 2,400 MHz; video card GTX1080Ti 11G. A camera and high-resolution smartphone were used for the sampling. The resolution was 16 million pixels.

### Image and Data Collection

**FIGURE 2:**
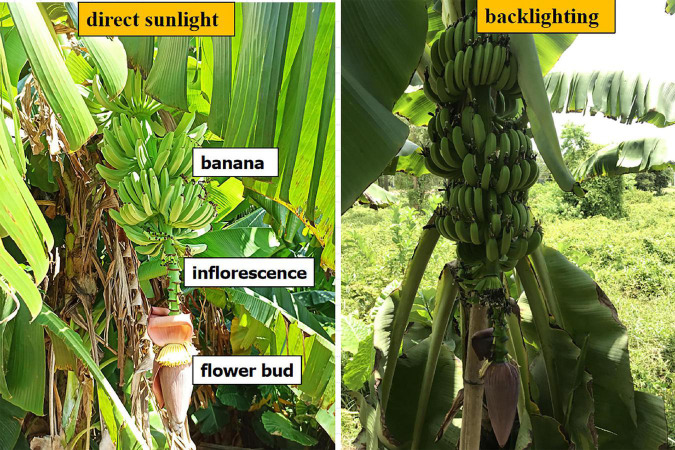
Samples of bananas, flower buds, and inflorescence axes under front-lighting and backlighting.

The sampling objects were banana fruits, flower buds, and inflorescence axes connecting the buds to the fruits (Figure 2). Each banana tree had only one bud, with the bud and the inflorescence axis pointing vertically downwards. When the fruits reach a particular stage of growth, the bud should be cut off from the inflorescence axis; this is known as bud cutting. Banana fruits, buds, and inflorescence axes are three types of targets with different features. The detection of these targets is influenced by the random shading of leaves or plants, background noises, and lighting. In this study, multi-target recognition was performed under different lighting conditions. The sampling strategy was to sample wherever there was a flower bud. The objective was the automatic recognition of flower buds and inflorescence axes to formulate the positioning decision for robotic bud picking. Besides, the multi-target sampling of bananas lays the basis for yield estimation and maturity assessment.

The experimental images were obtained in two batches. The photographs were taken on July 25, 2020, which was a sunny day, at the Lingnan Fresh Fruit Base of the Guangzhou Fruit World in Guangdong Province, China. The weather changed from sunny to cloudy from August 4, 2020 to August 5, 2020. The location was a banana orchard in Jiangmei, Guangdong Province. Multiple targets, including fruits, inflorescence axes and buds, were included in the photographs. Thus, the banana plants had multiple features that could be detected as targets in this study. During the sessions, 5,343 images were collected under different lighting conditions. Among them, 5,300 images were selected and subjected to annotation using an image annotation tool. A script was written for the automatic, random division of the samples into the training, validation, and testing sample sets. There were 4,800 images in the training set, 364 images in the validation set, and 685 images in the testing set, accounting for 90.60, 7, and 13%, respectively.

## Method and Algorithm Description

### Multi-Target Classification of Banana Fruits, Flower Buds, and Inflorescence Axes

Samples for the multi-target recognition of bananas are shown in Figure 2. Class annotation was performed for the original multi-target images obtained in the experiment. The script was written for automatic annotation to reduce the manual time. The size of the sampled images was set to 721 × 960 pixels. An image of random size was input and scaled until the w or h was 416 pixels. Then, the image was used as the network input. That is, the input was a three-channel RGB image with a size of 416 × 416 pixels.

### Multi-Scale Feature Fusion Method

**FIGURE 3:**
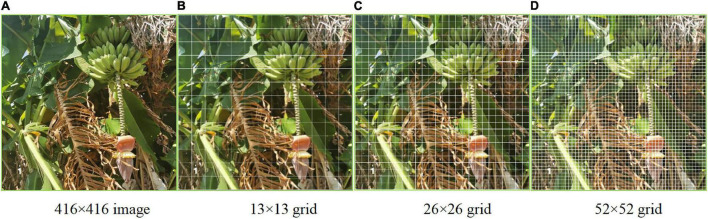
Multi-scale feature map of the bananas. **(A)** 416 × 416 image. **(B)** 13 × 13 grid. **(C)** 26 × 26 grid. **(D)** 52 × 52 grid.

For multi-target detection of bananas in which different features were recognized simultaneously, the prediction was done on multiple scales. The influence of resolution on the prediction is mainly determined by the resolution information, that is, the number of pixels (Figure 3). Logistic regression only applies to binary classification problems. While maintaining the accuracy, we designed a multi-label logistic classifier by modifying logistic regression to adapt to multi-classification problems. The new classifier utilized the Sigmoid function. If the confidence level for a bounding box was above 0.5 after feature extraction and after the Sigmoid function was constrained, it meant that the object surrounded by the bounding box was labeled correctly. In the YOLOv3 model belonging to the second type of method, the upsampling (like FPN) and data fusion algorithm were used to fuse banana images on three scales (13 × 13, 26 × 26, and 52 × 52). Then, target detection was performed separately on the multi-scale feature fusion map to improve the performance. The multi-scale fusion information about banana fruits, buds, and inflorescence axes is shown in Figure 3.

### YOLOv3 Network Architecture for Multi-Feature Targets

YOLOv3 utilized the feature extraction network part Darknet-53, which was composed of five residual blocks. This model borrowed from the residual neural network. The number of anchor boxes used in the algorithm changed from 5 in the original YOLOv2 model to 9. The size of the anchor boxes was calculated by applying k-means clustering to optimize the width and length of the actual target frame of the bananas (Redmon and Farhadi, 2017).

**FIGURE 4:**
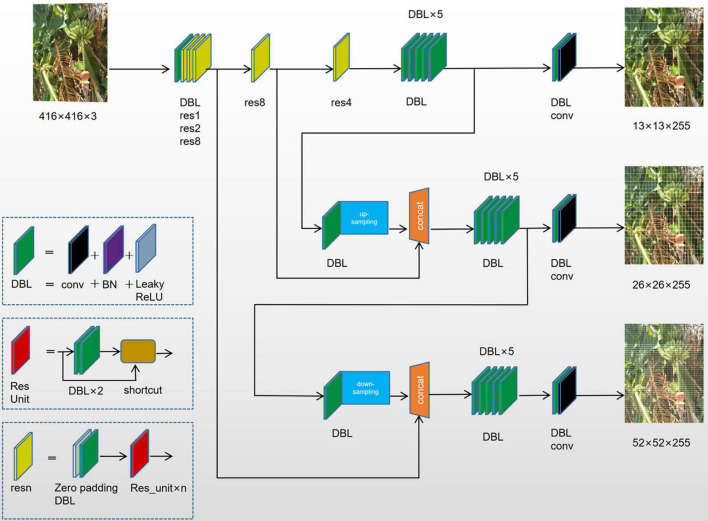
YOLOv3 network architecture for the multi-feature targets of banana plants.

The separate detection of each feature of the banana plants (inflorescence axes and buds) was performed using multi-scale feature fusion maps. This method could enhance the detection performance for targets of varying sizes and also the shaded ones. Besides, connections were introduced between the layers to strengthen the convergence performance. The multi-target detection of bananas was conducted in a complex field environment where noise interference abounded. The parameters of the existing YOLOv3 architecture still needed to be modified. Here, the loss function was modified and improved. The network architecture is shown in Figure 4.

### Loss Function

The loss function of YOLOv3 can affect the model convergence. It also serves as the basis for penalizing incorrect detection. The Sigmoid function was designed and used for the activation of the final output. Subsequently, SSE was used to calculate the final loss. However, the Sigmoid function has a saturation problem. Once the input falls within the saturation region, it approaches zero. As a result, the gradient nearly vanishes. If the error value calculated by using the squared error is very small, the network parameters can hardly be trained effectively (Lyu et al., 2019). One way to solve this problem is to introduce a cross-entropy loss function when the true value is either 0 or 1, as given by


(1) $$\text{Loss}=-\frac{1}{n}\sum_{x}\left(y*\ln\left(a\right)+\left(1-y\right)*\ln\left(1-a\right)\right)$$

where a is the output value of the error model after the introduction of a Sigmoid function. The YOLOv3 loss function consists of three parts, namely, location error (set to *l_1box*), classification error (*l_2obj*), and error of the confidence level (*l_3cls*). The squared error is considered as a function to reduce the cumulative error of the loss function and to mitigate the gradient vanishing when calculating the coordinate errors (location errors). If this error increases, the parameter gradient will increase. But when the error is very large, the parameter gradient decreases, leading to an uncertainty problem. Therefore, when calculating the confidence interval and the classification error, we introduced a cross-entropy loss function (Yin et al., 2020; Cao et al., 2021). Based on the initial YOLO loss function, the loss function was built using (6). Thus, the modified loss function has the following form:


(2) $$\begin{array}{c} Loss={\text{l}}_{1}box+l_{2}obj+l_{3}cls=\lambda_{cobox}\sum_{i=1}^{s^{2}}\sum_{\text{i}=0}^{B}{\text{I}}_{i,j}^{obj}\left(2-\left(w_{i}-hi\right)\right)\\ A_{wh}+\lambda_{cobox}\sum_{i=1}^{s^{2}}\sum_{i=0}^{B}{\text{I}}_{i,j}^{obj}{\text{A}}_{\text{xy}}+\lambda_{noobj}\sum_{i=1}^{s^{2}}\sum_{i=0}^{B}I_{i,j}^{obj}A_{xy}\\ +\lambda_{noobj}\sum_{i=1}^{s^{2}}\sum_{i=0}^{B}I_{i,j}^{noobj}C_{ii}+\lambda_{obj}\sum_{i=1}^{s^{2}}\sum_{i=0}^{B}I_{i,j}^{obj}C_{ii}\;\\ +\lambda_{class}\sum_{i=1}^{s^{2}}\sum_{i=0}^{B}I_{i,jc\in class}^{obj}\left[pi\left(c\right)\log\left({\text{p̌}}_{i}\left(c\right)\right)+{\text{D}}_{\text{p}}\right]\; \end{array}$$

which indicates whether the ${\text{I}}_{I,j}^{o\,b\,j}$ bounding box of the grid I and grid J network is responsible for detecting the banana targets. _cobox_ is used to improve the stability of the loss function and enhance the coordinate loss prediction of the bounding box. is the weight coefficient of the coordinate error model. _obj_,_noobj_ are for the trade-off between the positive and negative samples, representing the weight coefficients for the error of the confidence level with the target included and excluded, respectively. λ_*class*_ is the weight coefficient for the classification error. w_i_*h*_*i*_ are the width and height of the real target frame of bananas, respectively; *C_i* is the confidence level of the real banana target; *p_i* is the class probability prediction. In the squared error term, let ${\text{A}}_{\text{wh}}=(w_{i}-{\hat{w_{i})}}^{2}+{\left(h_{i}-\hat{h_{i}}\right)}^{2};A_{x\,y}={\left(x_{i}-\hat{y_{i}}\right)}^{2};C_{i\,i}=\hat{C_{i}}\log\left(C_{i}\right)+\left(1-\hat{C_{i}}\right)\log\left(1-C_{i}\right);D_{p}=\left(1-\hat{p_{i}}\left(c\right)\right)\text{log}(1-p_{i}\left(c\right)$.

Multi-target detection of bananas covered the fruits, inflorescence axes, and buds. Therefore, unlike usually, softmax was not chosen for predicting the class labels. Instead, logistic regression was used to predict the class. The function for realizing multi-target prediction with multi-scale feature fusion is known as the logistic function, given by


(3) $$f_{x}=\frac{1}{1+e^{-1}}$$

### Modification of the YOLOv3 Model Based on Clustering Optimization and Model Evaluation

A trade-off was considered between the elapsed time and prediction. First, some parameters of the YOLOv3 model were optimized based on environmental features and the biological features of bananas as the multi-feature target. During model training, the epoch parameters were 100 iterations and batch_size 32, which increased the elapsed time and memory consumption. While ensuring precision, the epoch, number of iterations, and batch_size were set to 50, 39, and 8, respectively. The experimental results showed that when the number of iterations was approximately 50, the loss function curve tended to stabilize. The YOLOv3 model with optimized parameters for multi-target recognition of bananas was known as the modified YOLOv3 thereafter.

Based on the above, the dimensionality of the target candidate frame in the YOLOv3 model was subject to clustering optimization to optimize the YOLOv3 model and improve the precision. The YOLOv3 had default values for the number of target candidate frames and height-to-width ratio and hence, enjoyed universality to a certain degree. However, the YOLOv3 still needs to be optimized when applied to the multi-target detection of bananas in a complex field environment and changing lighting conditions. Here, the YOLOv3 model was optimized using the fusion clustering algorithm (known as YOLOv3 based on clustering optimization). Clustering was performed using the k-means clustering and training dataset. The number of target candidate frames, height, and width fit for the prediction were updated. The parameters of the multi-target candidate frame are shown in Table 1. We conducted a multi-target recognition experiment using the YOLOv3 model based on clustering optimization under different lighting conditions.

**TABLE 1:** Parameters of candidate frames for multiple features of bananas.

Serial No.	1	2	3	4	5	6	7	8	9
Height of the new candidate frame	23	25	28	31	43	51	66	169	172
Width of the new candidate frame	31	53	81	148	67	87	111	233	158
Height of the original candidate frame	10	16	33	30	62	59	116	156	373
Width of the original candidate frame	13	30	23	61	45	119	90	198	326

To assess the generalization ability of the deep learning network and optimize the model stepwise, we determined the precision (P_*re*_), recall, F1-score and Matthews Correlation Coefficient (MCC) as precision measure of the binary classification model. The calculation formulae are shown in Eqs. (4)–(6):


(4) $$P_{re}=\frac{T_{p}}{T_{p}+F_{p}}\times 100\%$$

(5) $$Recall=\frac{T_{p}}{T_{p}+F_{n}}\times 100\%$$

(6) $$F_{1}=\frac{2\times P_{re}\times Recall}{P_{re}+Recall}\times 100\%$$

(7) $$MCC=\frac{T_{p}\times T_{n}-T_{p}\times F_{n}}{\sqrt{\left(T_{p}+F_{p}\right)\times \left(T_{p}+F_{n}\right)\times \left(T_{n}+F_{p}\right)\times \left(T_{n}+F_{n}\right)}}$$

where is the true positive *T_p* is the false positive, and the false *F_p* is preserved; F_n_ is the false negative, and the true is removed.

During the training experiment, the full data set of the multi-target recognition of bananas was run once (epochs). For each epoch, one group of precision and recall was obtained. By setting different thresholds of the modified YOLOv3 model, several groups of precision and recall were obtained, and a PR curve was drawn. The area under the curve was the average precision (AP).

## Calculation Method for the Cutting Point

Geometrically, the flower buds are inverted cones with basic symmetry. The cutting point of the bud is located on the inflorescence axis. The midpoint of the bounding rectangle of the inflorescence axis can be easily found when the bud and the inflorescence axis point vertically downwards. However, some buds and inflorescence axes do not point vertically downwards but at an angle δ with respect to the vertical direction. In this case, finding the midpoint of the bounding rectangle of the inflorescence axis to position the cutting point may lead to mistakes.

**FIGURE 5:**
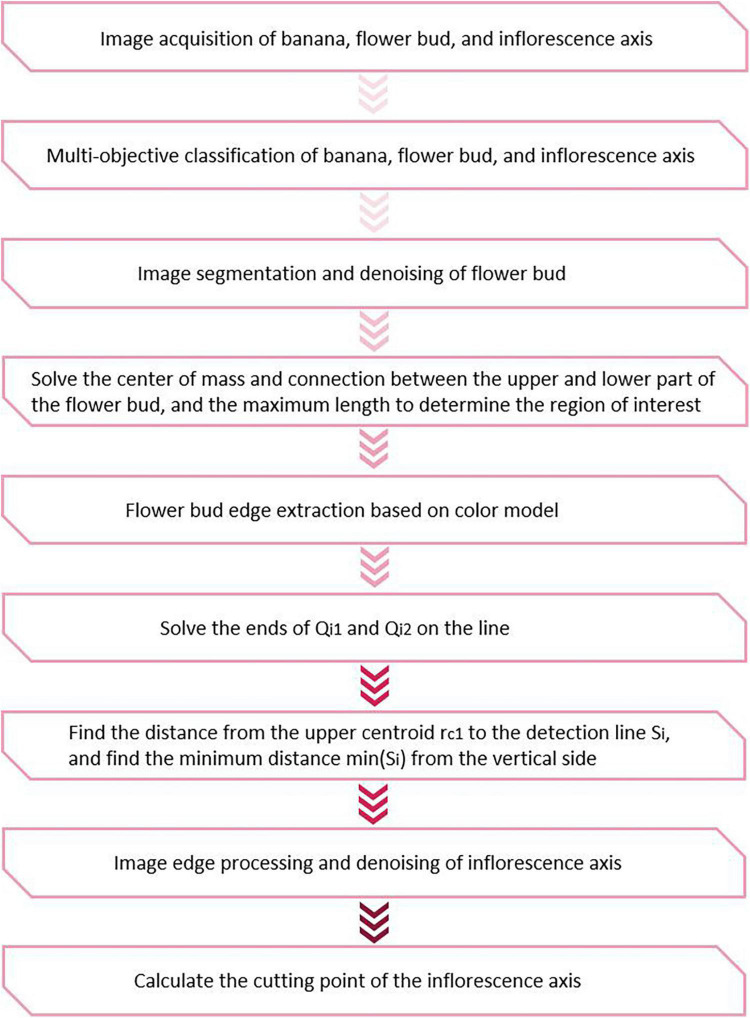
Flowchart of cutting point positioning.

Without loss of generality, the cutting point should be calculated by determining the centerline when the bud is at an angle of δ. First, multi-target classification was done using the YOLOv3 model based on clustering optimization. The image edges of the buds and inflorescence axes were determined using the edge algorithm. The centroid and the center of geometric symmetry were solved based on the symmetry of the bud. Next, the cutting point in the inflorescence axis was calculated using the geometric method. This method first determined the distance from all the detected straight lines to the centroid. Then, the straight line with the shortest distance was chosen by imposing the minimum constraint on the distance from the point to the line. This is the line along which the inflorescence axis runs. The midpoint of this line is chosen based on the coordinates and treated as the cutting point. The spatial coordinates of this cutting point forms the basis for configuring the parameters of tool posture in the robot actuator. The schematic of the cutting point positioning algorithm is shown in Figure 5.

### Image Segmentation

Image segmentation for flower buds and inflorescence axes is the basis of the cutting point positioning algorithm. Multi-target classification and recognition of banana fruits, flower buds, and inflorescence axes were first performed for noise reduction. However, some bud images might contain green or dry leaves, fruit branches, and lighting noises. The bud color differs from the color of a few green or dry leaves. Here, the bud edges were extracted by extracting the color components and by Otsu’s binarization.

**FIGURE 6:**
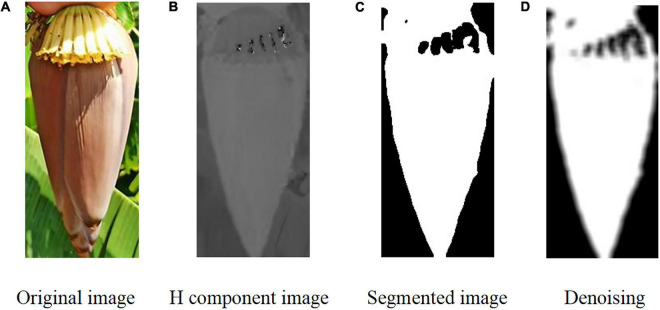
Image segmentation process of the banana buds. **(A)** Original image. **(B)** H component image. **(C)** Segmented image. **(D)** Denoising.

Mathematical morphology is applicable to the denoising of complex images and image restoration due to its intuitiveness and suitability for processing geometrical structures. The opening operation can inhibit positive impulse noise in the bud signals, while the closing operation can inhibit the negative impulse noise. To remove the positive and negative noise from the signals simultaneously, we combined the opening and closing operations to form a morphology filter. Noise in the binary images of the buds were mainly composed of the surrounding noise blocks and the noise holes inside. The opening operation was adopted to remove the noise surrounding the buds, and the closing operation was for removing the noise holes inside. That is, set A was closed using the structure element B. Denoising was performed using the morphological method. However, the black noise within the contour of the bud became enhanced (Figure 6).

After image segmentation, some small curled petals might be separated from the overall contour of the bud under certain conditions. However, these petals were of negligible size. To reduce the error in the computation of the image centroid, we proposed extracting the maximum connected region from the main contour of the bud. In this study, 50 sample images were used for cutting point positioning on the inflorescence axis. Given the large sample size, the conventional denoising approach usually has a low efficiency as denoising is performed for one image at a time. Here, denoising was performed using the batch processing method.

### Solving the Image Centroid

After extracting the maximum connected region, a binary image was obtained for the bud region. The pixel value of this region was set to 1 (white), while the remaining was set at 0 (black). The centroid coordinates of the bud were estimated (Luo et al., 2018) using the formula below according to the definition of the moment of the image centroid:


(8) $$\left\{ \begin{array}{c} x_{ci}=\sum xf\left(x,y\right)/\sum f\left(x,y\right)\\ y_{ci}=\sum yf\left(x,y\right)/\sum f\left(x,y\right) \end{array}\right.$$

where x_ci_,*y*_*ci*_ are the centroid coordinates of the upper and lower parts of the bud, respectively; *x*,*y* are the pixel coordinates; *f*(*x*,*y*) is the pixel value of the binary image at point (x,y).

**FIGURE 7:**
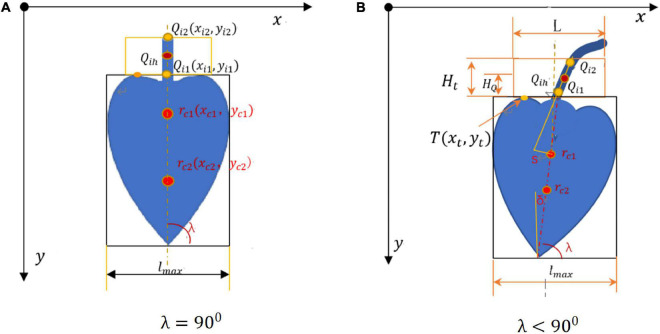
Schematic for solving image centroid and cutting point. **(A)** = 90^0^. **(B)** = 90^0^.

After binarization, the image in the H channel was divided into the upper and lower parts. The pixel location information was estimated for each target. It was determined whether the pixel point fell within the bud region. The pixel information was estimated and the centroids in the upper and lower parts were determined. The centroid *r*_*c*1_(*x*_*c*1_,*y*_*c*1_) in the upper part and the centroid *r*_*c*2_(*x*_*c*2_,*y*_*c*2_) in the lower part were connected to find the centerline of the bud. The image centroid of the banana buds is shown in Figure 7. After obtaining the centroid coordinates, the minimum bounding rectangle was found for the bud region by fitting. When λ = 90^0^, = 0, the bud and the inflorescence axis point vertically downwards and are symmetric. When λ < 90^0^, the bud and the inflorescence axis are at an angle of δ with respect to vertical direction, as shown in Figure 7B.

**FIGURE 8:**
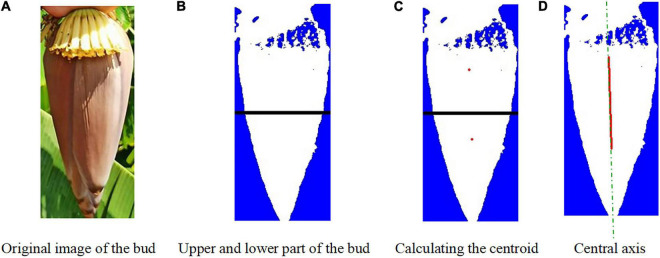
Image centroid calculation for the buds. **(A)** Original image of the bud. **(B)** Upper and lower part of the bud. **(C)** Calculating the centroid. **(D)** Central axis.

*l*_*max*_ is the maximum width of the bud contour; *s* is the distance from the detected straight line to the centroid; *T*(x_t_,*y*_*t*_) is the highest point on the bud edge; *Q*_*i1*_ and *Q*_*i2*_ are end points of the detected line segment; *H_t* is the height of our interest in the inflorescence axis; *Q*_*ih*_ is the cutting point in the inflorescence point, with a distance of *H_Q*. Based on the principles above, the image centroid and central axis were found for the buds, as shown in Figure 8.

### Geometric Calculation of the Position of the Cutting Point in the Bud

According to the growth features of the buds and inflorescence axes and the principles of geometric method, we constructed a schematic diagram for the calculation of the cutting point. First, the bounding rectangle of the inflorescence axis was found. Then, the inflorescence axis information and growth direction were extracted. Finally, the coordinates of the optimal cutting point were determined.

**FIGURE 9:**
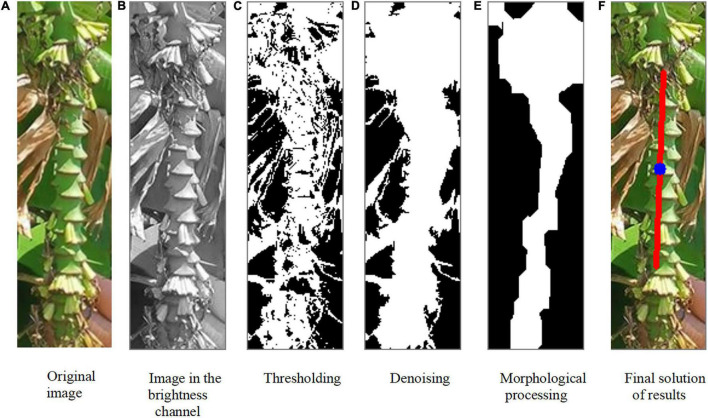
Inflorescence axis extraction and growth direction calculation. **(A)** Original image. **(B)** Image in the brightness channel. **(C)** Threshholding. **(D)** Denoising. **(E)** Morphological processing. **(F)** Final solution of results.

Due to shading and color interference, it was difficult to extract the inflorescence axis. In Figure 9A, the inflorescence axis and the banana leaves in the background are similar in color. Therefore, it is difficult to extract the inflorescence axis region by setting a specific hue. Besides, the inflorescence axis itself has a complex geometric shape. Under a particular illumination, shadows will appear on the boundaries of the inflorescence axis, causing considerable interference in the binary image. Here, the inflorescence axis was treated as a slender cylinder in axial symmetry, and the region was extracted by image processing. Thus, the problem of extracting inflorescence axis information was converted into a problem of extracting the binary mask of the inflorescence axis.

The images where converted into the HSV coordinate system; the HSV channels were observed, and the V channel (Figure 9B) showed the best separation of the inflorescence axis from the background. Thus, image segmentation was primarily performed in the V channel. To remove the problems of random colors and uneven brightness, we subtracted the values in the V and H channels after histogram equalization. This was followed by a phase inversion. After this procedure, only the portion with higher brightness remained as noise (Figure 9C). The boundaries of the inflorescence axis in the original image were blurred. We ran the contour-finding algorithm for all the homochromatic simply connected regions in Figure 9C. The regions below the threshold within the contour were eliminated. In this way, nearly all the noise was removed while maximally preserving the boundary features of the inflorescence axis (Figure 9D).

The following assumption was made within the bounding rectangle of the inflorescence axis to eliminate the influence of the remaining irrelevant area and boundary irregularity of the inflorescence axis: The inflorescence axis had the largest area ratio. Figure 9D was further subject to morphological processing, such as expansion and corrosion, to obtain Figure 9E. Then Figure 9E was solved to calculate the growth direction of the inflorescence axis (Figure 9F).

As to the bounding rectangle of the inflorescence axis, the boundaries were determined by finding the pixel pair. First, the boundary coordinates of the inflorescence axis were estimated and clustered. The outliers were eliminated by using the clustering-based denoising algorithm. The feature boundary line was fitted. The center of the inflorescence axis was found based on the mean diameter or pixels of the inflorescence axis.

The pixel coordinates of the two end pointsQ_*i1*_, Q_*i2*_ in the line segment of the inflorescence axis are (x_*i1*_, *y*_*i*1_) and (x_*i2*_, *y*_*i*2_), respectively. The two endpoints are connected by a straight line:


(9) $${\text{l}}_{\text{i}}\left(x,y\right)=\left(x-x_{i1}\right)\left(y_{i2}-y_{i1}\right)-\left(y-y_{i1}\right)\left(x_{i2}-x_{i1}\right)$$

The centroid in the upper part of the bud and the center of the bud were already known. The bud was basically symmetrical. The inflorescence axis is usually above the centroid of the bud. When the bud and the inflorescence axis point vertically downwards, the centerline of the bud overlaps with the axis of the inflorescence axis (Figure 7A). The extension of the axial line of the inflorescence axis passes through the centroid of the bud (Luo et al., 2018). But in a real orchard, a few buds do not point vertically downwards but at an angle of δ (Figure 7B). Here, the minimum constraint function *min*(*S*_*i*_) for the distance from the point to the line was solved to find the straight line segment where the cutting point was located. The midpoint of this straight line segment *Q*_*ih*_ was considered the cutting point (Figure 9F).

By analogy with the method of solving the distance from the point to the straight line segment, we found the distance from each straight line segment to the centroid *S_i*:


(10) $$S_{i}=min\frac{\left(x_{c1}-x_{i1}\right)\left(y_{i2}-y_{i1}\right)-\left(y_{c}-y_{i1}\right)\left(x_{i2}-x_{i1}\right)}{\sqrt{{\left(y_{i2}-y_{i1}\right)}^{2}+{\left(x_{i2}-x_{i1}\right)}^{2}}}$$

## Experimental Results and Analysis

### Classification and Recognition of Banana Fruits, Flower Buds, and Inflorescence Axes

The modified YOLOv3 model was trained using the training set and subsequently verified using the validation set. Features were repeatedly extracted from the trunk layer and the detection layer on multiple scales to improve the efficiency of the network in detecting tiny objects. A comparison was made between the modified YOLOv3 model, YOLOv4 model, and Faster R-CNN. The values in the brackets are the precision.

**FIGURE 10:**
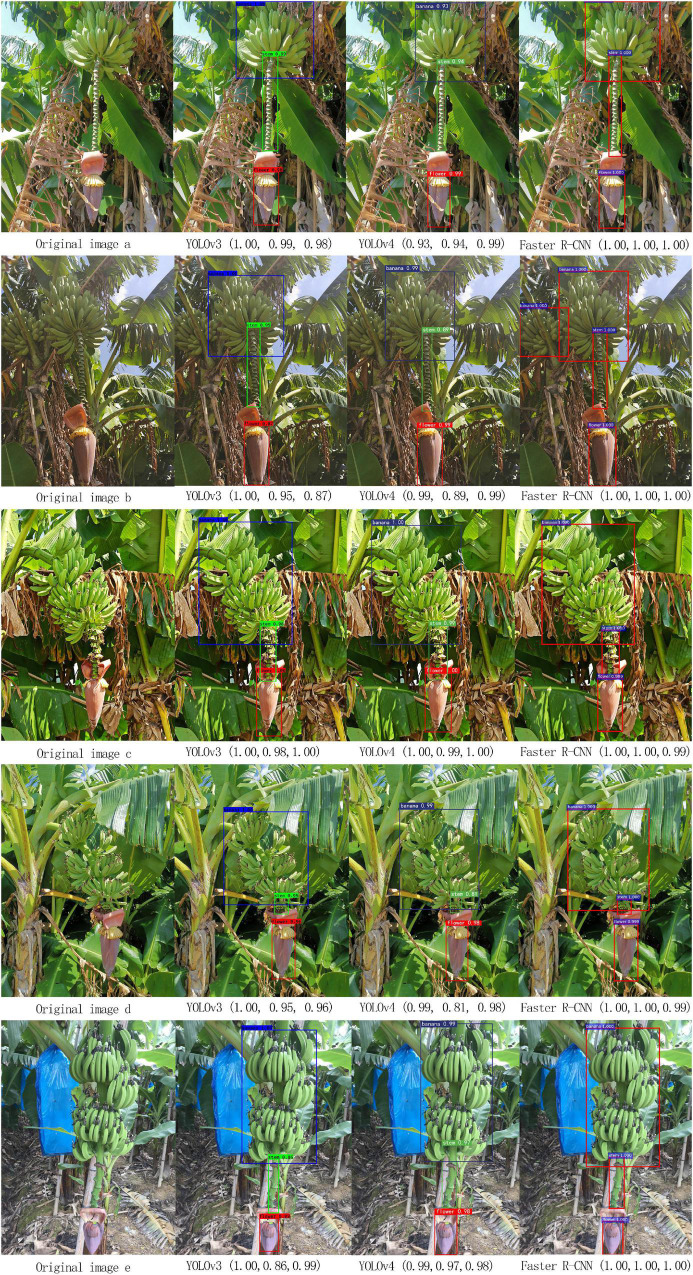
Comparison of the multi-target recognition results of bananas.

As shown in Figure 10, the precision was higher for banana fruits and buds. YOLOv3 had a higher precision for detecting the inflorescence axis, which was comparable to that of the faster R-CNN. However, when the inflorescence axis was very short, a part of it was blocked by the petals growing upwards. Besides, the short inflorescence axis could hardly be separated from the background. Apparently, lighting had a large impact on the precision of the YOLOv3 model.

Comparison of the precision and elapsed time for the modified YOLOv3, faster R-CNN, and YOLOv4 in multi-target detection is shown in Table 2. The average elapsed time for each image in the sample set is denoted by t.

**TABLE 2:** Comparison of the multi-target detection results between the YOLOv3 model, YOLOv3 model based on clustering optimization, R-CNN model and YOLOv4 model (238 images).

Model	Flower buds		Banana		Inflorescence axis		mAP	*t* (s)
	AP	Recall	AP	Recall	AP	Recall		
Faster-R-CNN	98%	99.90%	99%	99%	90%	90.30%	95.80%	0.43
YOLOv3	98%	97.83%	96%	95.95%	85%	85.07%	92.95%	0.24
Modified YOLOv3	97%	97.90%	94%	93.60%	88%	90%	92.98%	0.24
YOLOv4	98%	98.64%	96%	96.76%	86%	88.21%	93.46%	0.20

Table 2 shows that the mAP of YOLOv3 and clustering optimization-based YOLOv3 was 92.95 and 92.98%, respectively, for the multi-target detection of bananas. The average elapsed time for the detection of each image was 0.24 s. Table 2 shows that the mAP of the YOLOv4 model was 93.46% for the multi-target detection of bananas. The average elapsed time for the detection of each image was 0.2 s. The mAP of Faster R-CNN was 95.80% for the multi-target detection of bananas. The average elapsed time for the detection was 0.43 s per image.

To account for the influence of lighting on multi-target recognition, 1,062 and 1,072 images of banana fruits, buds, and inflorescence axes in front-lighting and backlighting conditions, respectively, were used for the recognition experiment.

The results showed that under the front-lighting condition, YOLOv3 based on clustering optimization had the highest mAP, which was 97.90%, followed by Faster-R-CNN. Both had a precision above 97.00%. Under the backlighting condition, Faster-R-CNN had the highest precision of 97.47%, followed by YOLOv3 based on clustering optimization. The overall average recall was 97.55% with Faster-R-CNN vs. 95.50% with YOLOv3 based on clustering optimization. In the front-lighting condition, both models had a comparable recall. But in the backlighting condition, Faster-R-CNN had a higher recall.

Taken together, YOLOv3 based on clustering optimization performed better in both front-lighting and backlighting conditions with a higher recall. The modified YOLOv3 model based on clustering optimization performed well with a good balance between speed and precision under both the front-lighting and backlighting conditions. During the robotic bud cutting and picking operation, the modified YOLOv3 based on clustering optimization can preferably be chosen for positioning in the front-lighting condition.

### Positioning of the Banana Buds and Cutting Point in the Inflorescence Axes

Thirty groups of samples were chosen for the cutting point positioning experiment. First, the cutting point was positioned manually in the inflorescence axis. Next, the proposed algorithm was run for calculation and comparison, and the error was estimated.

Let the optimal cutting point positioned manually on the inflorescence axis be *M*_*ih*_. The calculated cutting point is *Q*_*ih*_. Thus, the error Δ is given by:


(11) $$\Delta_{\text{x}}=\left|{\text{X}}_{\text{i}}-x_{i}\right|$$

(12) $$\Delta \text{y}=\left|{\text{Y}}_{\text{i}}-y_{i}\right|$$

where *X*_*i*_,*Y*_*i*_ are the pixel coordinates of the i row and the i column in the pixel region for the optimal cutting point manually determined in the inflorescence axis, respectively. *x*_*i*_,*y*_*i*_ are the pixel coordinates of the i row and the i column for the calculated cutting point in the inflorescence axis, respectively.

The pixel scope of the optimal cutting point (20 ± 15 pixels × 65 ± 30 pixels) was manually set up. The optimal cutting point was located along the centerline of the inflorescence axis (20 ± 15 pixels × 65 ± 30 pixels). The pixel positioning errors calculated for the 60 images are shown in Table 3. X and Y are the pixel scopes of the optimal cutting point; x and y are the coordinates of the calculated cutting point; *e_x* is the pixel positioning error along the row direction; *e_y* is the pixel positioning error along the column direction; *e* is the overall pixel positioning error.

**TABLE 3:** Comparison of the multi-target detection results between the YOLOv3 model, YOLOv3 model based on clustering optimization, R-CNN model and YOLOv4 under front-lighting and backlighting conditions.

Model	AP for fruits		AP for flower buds		AP for fruit axes		Mean average precision (mAP)	
	Front-lighting	Backlighting	Front-lighting	Backlighting	Front-lighting	Backlighting	Front-lighting	Backlighting
Faster-R-CNN	100%	100%	100%	100%	93%	93%	97.64%	97.47%
YOLOV3 based on parameter optimization	100%	95%	94%	92%	84%	66%	92.63%	84.38%
YOLOV3 based on clustering optimization	98%	94%	100%	97%	95%	82%	97.9%	91.11%
YOLOV4	100%	96%	96%	92%	89%	74%	95.30%	87.37%

**TABLE 3–1:** Comparison table of cutting point and pixel positioning error under two types of lighting.

Image Image frame	Scope of the optimal		Calculated cutting		Error between the optimal and		
	cutting point/pixel		point/pixel		the calculated cutting point/pixel		
	X	Y	x	y	e_x_	e_y_	e
Front-lighting on sunny days	47	147	44	145	3	2	4
Backlighting on sunny days	85	202	88	206	3	4	5

**TABLE 3–2:** Statistical table of cutting point positioning under two types of lighting.

Lighting conditions	Original image/frame	Number of images meeting the positioning requirements/frame	Percentage of images meeting the positioning requirements	Average elapse time
Front-lighting on sunny days	30	28	93%	439
Backlighting on sunny days	30	27	90%	448

As shown by the cutting point positioning errors for the buds and inflorescence axes, the error was kept below 15 pixels for 55 images. On these images, the cutting point was positioned along the edge of the inflorescence axis. The error was above 15 pixels for five images, where the cutting point was positioned outside the inflorescence axis. The positioning error along the Y-direction was significantly lower than that along the X-direction. This was probably because the mask generated by the morphological processing of shadows on the edge of the inflorescence axis shifted in the X-direction.

When detecting the rectangular region associated with the inflorescence axis, the cutting point might be mistakenly positioned at the edge of the axis. However, the optimal cutting point must be located on the centerline of the inflorescence axis. The positioning error can be estimated as the radius of the fruit stem. We have developed a clamping and cutting mechanism for the end actuator based on fault tolerance analysis (Zou et al., 2016). This mechanism can compensate for the pixel positioning error of the cutting point in the X (row) direction and identify the cutting point.

The previous algorithms can only get better results when facing fruits with simple outline shape, but cannot get better classification results when facing fruits with complex and irregular or fruits with complex growing environment (such as bananas). The algorithm proposed in this study can further calculate the detected target on the basis of deep learning, and has achieved high detection accuracy in both bright and backlight environments, and has high robustness in cooperation with the end effector based on fault-tolerant design developed by us.

## Conclusion

This study focused on the multi-target recognition of banana fruits, buds, and inflorescence axes in a complex orchard environment, and proposed YOLOv3 model and edge detection algorithm based on cluster optimization, and constructed a calculation model of flower bud cutting point. Experiments showed that the mAP and speed of modified YOLOv3 and YOLOv4 were satisfactory. The precision was 92.98 and 93.46%, respectively. The average time for field detection of each image was 0.24 and 0.2 s, respectively. The accuracy of the improved YOLOv3 is 97.90 and 91.11% under the conditions of front-lighting and backlighting conditions, respectively. The improved YOLOv3 had better performance and higher recall rate, and had achieved a good balance between speed and accuracy. Under the conditions of front-lighting and backlighting, the total pixel positioning errors between the calculated optimal cutting point and the manually determined optimal cutting point in flower bud were 4 and 5 pixels, respectively. The proportion of images meeting the positioning requirements was 93 and 90%, respectively. The experiments showed that the proposed algorithm could satisfy the requirements for recognition performance and comprehensive performance in the cutting point positioning process.

Multi-target classification and recognition of bananas from images potentially offer data support for the yield estimation of bananas. In the present study, we performed multi-target detection of bananas using monocular vision and by calculating the cutting point on the xy-plane. We recommend stereoscopic vision to obtain the 3D spatial information required for the detection. Another important research task related to the smart banana orchard operation is the robotic recognition of spatial coordinates of the inflorescence axis based on stereoscopic vision and the robot obstacle avoidance and cutting behavior.

## Data Availability Statement

The original contributions presented in the study are included in the article/supplementary material, further inquiries can be directed to the corresponding author/s.

## Author Contributions

JD designed the experiments. YY and SC carried out the experiments. PA and FW analyzed experimental results with improved algorithms. FW wrote the manuscript. JD and ZY supervised and revised the manuscript. All authors contributed to the article and approved the submitted version.

## Conflict of Interest

The authors declare that the research was conducted in the absence of any commercial or financial relationships that could be construed as a potential conflict of interest.

## Publisher’s Note

All claims expressed in this article are solely those of the authors and do not necessarily represent those of their affiliated organizations, or those of the publisher, the editors and the reviewers. Any product that may be evaluated in this article, or claim that may be made by its manufacturer, is not guaranteed or endorsed by the publisher.
